# Longitudinal variations in thermospheric parameters under summer noontime conditions inferred from ionospheric observations: A comparison with empirical models

**DOI:** 10.1038/s41598-019-49255-1

**Published:** 2019-09-04

**Authors:** Andrey V. Mikhailov, Loredana Perrone

**Affiliations:** 1 Pushkov Institute of Terrestrial Magnetism, Ionosphere and Radio Wave Propagation (IZMIRAN), Moscow, Russia; 2 Istituto Nazionale di Geofisica e Vulcanologia (INGV), Rome, Italy

**Keywords:** Planetary science, Astronomy and planetary science

## Abstract

Longitudinal variations in the thermospheric neutral composition ([O] and [N_2_]) and exospheric temperature Tex have been inferred from June monthly median noontime f_o_F_1_ and f_o_F_2_ observations at mid-latitudes to check for consistency with empirical MSIS models. In general, a similarity in longitudinal variations has been demonstrated, and this is interesting, as similar variations were obtained with very different methods and different data sources. Both inferred and MSISE-00 modelled height-integrated O/N_2_ ratios are comparable to TIMED/GUVI observations only under solar minimum conditions but differ substantially under high solar activity. The retrieved height-integrated O/N_2_ ratio longitudinal variations are small (∼15%) in comparison to the observed N_m_F_2_ variations under high solar activity. The height-integrated O/N_2_ ratio cannot be incorporated into the F_2_-layer formation mechanism; therefore, such observations cannot be used for any quantitative interpretation of N_m_F_2_ variations.

## Introduction

Ionospheric parameters during the daytime reflect the state of the surrounding thermosphere and the intensity of incident solar extreme ultraviolet (EUV) radiation; therefore, thermospheric and ionospheric parameters should demonstrate consistent spatial variations. However, historically, global ionospheric IRI^1^ and thermospheric models, for instance, MSISE-00^2^ empirical model, have been developed independently of each other, and there is no certainty in their consistency. A direct use of the MSIS model to calculate electron concentration in the ionospheric F region may give unsatisfactory results, and model parameters must be corrected to fit the observed N_m_F_2_ (electron concentration in the F_2_-layer maximum) under specific geophysical conditions^3–7^. Existing global first-principle (physical) models cannot yet compete with empirical models for many reasons^8,9^ and cannot answer the question of consistency between thermospheric models and ionospheric observations.

A recently developed method^10^ solving an inverse problem of aeronomy allows us to retrieve a consistent set of main aeronomic parameters responsible for the formation of the daytime mid-latitude ionospheric F-region. Using observed near noontime f_o_F_1_ and f_o_F_2_ (critical frequencies of the F_1_ and F_2_ layers, respectively, related to an electron concentration of Ne = 1.24 × 10^4^ fo^2^) and the standard indices of solar (F_10.7_) and geomagnetic (Ap) activity as the input information, the method^10^ provides a neutral composition ([O], [O_2_], and [N_2_]); exospheric temperature Tex; vertical plasma drift W, which may be converted into effective thermospheric meridional wind Vnx; and total solar EUV flux, with λ ≤ 1050 Å. The inferred aeronomic parameters determine plasma production, as well as its dynamics and recombination at F-region heights. Thus, by solving the inverse problem of aeronomy, we have an opportunity (via the inferred thermospheric parameters) to check the consistency between the observed longitudinal variations in ionospheric parameters (f_o_F_1_ and f_o_F_2_) and modern empirical thermospheric models. Unlike recent analyses of longitudinal variations dealing with integrated thermospheric characteristics, such as neutral gas density^11^ or height-integrated O/N_2_ ratio^12^, the proposed method provides individual thermospheric parameters ([O], [O_2_], [N_2_], and T_ex_). The electron concentration in the maximum of the F_2_-layer (N_m_F_2_) depends on individual [O] and [N_2_] concentrations rather than on the height-integrated O/N_2_ ratio, as is suggested in some publications^13^. In the beginning of the space era, thermospheric neutral composition ([O] and [N_2_]) was measured with mass- spectrometers, and these two species were shown to demonstrate different spatial variations^14^.

The aims of the paper may be formulated as follows:

- To analyse longitudinal variations in available noontime monthly median f_o_F_1_ and f_o_F_2_ observations for June under solar minimum and maximum conditions;
- To retrieve thermospheric parameters from f_o_F_1_ and f_o_F_2_ observations and to analyse their longitudinal variations in comparison with the empirical thermospheric models to check the consistency between them;
- To discuss the physical mechanism of the longitudinal variations in thermospheric and ionospheric parameters under June noontime conditions while considering the inferred neutral composition and recent height-integrated O/N_2_ ratio observations.

## Method

The method used in our analysis was described in a previous paper^10^. It is based on solving an inverse problem of aeronomy. The idea is to use routine ground-based f_o_F_1_ (or f_0180_ - plasma frequency at a 180 km height) and f_o_F_2_ near-noontime observations to find a consistent set of main aeronomic parameters responsible for the F-region formation under given geophysical conditions. The method has two versions that are used depending on the available input information. As long as we consider historical monthly median ionospheric observations, only summer f_o_F_1_ data are available, and we use June f_o_F_1_ observations when the F_1_ layer is distinct on ionograms and gaps in the data are practically absent. Historical monthly median electron density profiles Ne(h) used to read f_0180_ are absent. Daytime (10–14 LT) monthly median N_m_F_2_ and N_m_F_1_ observed by the worldwide ground-based ionosonde network in the Northern Hemisphere were used in our analysis. Such observations are available for 50–70 years at some stations.

By solving continuity equations for the main ionospheric ions and applying the method of multi-parametric optimization^15^, it is possible to fit the calculated N_m_F_2_ and N_m_F_1_ to the observed ones and to infer factors for the MSIS-86 model exospheric temperature Tex, neutral composition ([O], [O_2_], and [N_2_]), and the total solar ionizing EUV flux with λ ≤ 1050 Å from the model^16^. Under known neutral composition and temperature, the vertical plasma drift W can be obtained by fitting the calculated N_m_F_2_ to the observed one. In fact, all aeronomic parameters are found simultaneously in the iterations. The method was tested using CHAMP/STAR neutral gas density observations under various geophysical conditions, and it was shown to demonstrate advantages over modern empirical thermospheric models^10^.

## Results

An inspection of available simultaneous f_o_F_1_ and f_o_F_2_ June noontime observations over the Northern Hemisphere has shown that the largest amount of data was available in 1975, 1976, 1985, and 1986 for the solar minimum and in 1969, 1970, 1980, and 1981 for the solar maximum. Observations at 26 mid-latitude stations (http://spidr.ngdc.noaa.gov/spidr/) were used in our analysis (Table 1).

**Table 1:** Stations with available June monthly median f_o_F_1_ and f_o_F_2_ observations used in the analysis. Geographic latitudes, longitudes and magnetic latitudes of the stations are given.

Station	Lat, N deg	Lon, E deg	Mag. Lat deg	Station	Lat, N deg	Lon, E deg	Mag. Lat deg
Adak	51.9	183.4	47.5	Ottawa	45.4	284.1	56.3
Alma-Ata	43.2	76.9	33.3	Petersburg	60.0	30.7	56.0
Boulder	40.0	254.7	48.7	Point Arg	35.6	239.4	42.1
Ekaterinburg	56.7	58.6	48.6	Rome	41.9	12.5	42.0
Goosebay	53.3	299.6	64.1	Rostov	47.2	39.7	42.2
Gorky	56.1	44.2	50.0	Juliusruh	54.6	13.4	54.3
Irkutsk	52.5	104.0	41.2	Slough	51.5	359.4	53.8
Kaliningrad	54.7	20.6	52.7	St. Johns	47.6	307.3	57.9
Karaganda	49.8	73.0	40.2	Tomsk	56.5	84.9	45.9
Kiev	50.5	30.5	46.9	Tunguska	61.6	90.0	50.7
Kokubunji	35.7	139.5	25.7	Wakkanai	45.4	141.7	35.5
Magadan	60.1	151.0	50.9	Winnipeg	49.8	265.6	59.6
Moscow	55.5	37.3	50.6	Yakutsk	62.0	129.6	51.2

Observations were grouped by years with solar minima and maxima. Before this grouping, the observed f_o_F_1_ and f_o_F_2_ were reduced to the same latitude of 50°N and the same level of solar activity using the internal structure of the IRI model. The IRI model dependences of f_o_F_1_ and f_o_F_2_ on coordinates and solar activity were used for this reduction. The input index of solar activity to the IRI is a 12-month running mean sunspot number R_12_ or a 12-month running mean index F_10.7_ (F_12_), which is averaged for June over the years with a solar minimum R_12_ = 15 (F_12_ = 75) and for the years with a solar maximum R_12_ = 125 (F_12_ = 177).

**Figure 1:**
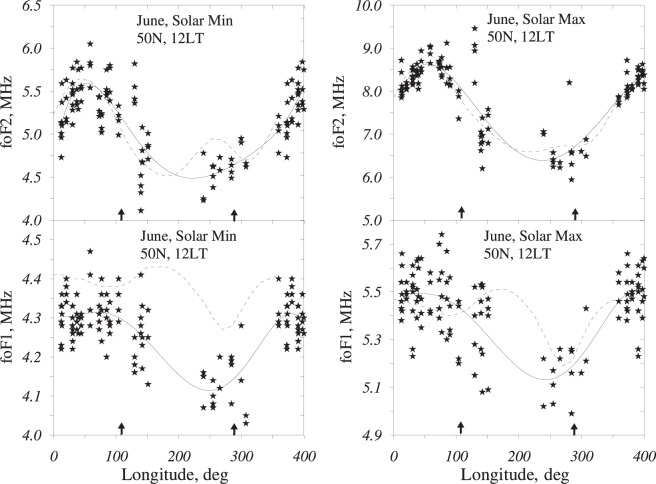
Longitudinal variations in reduced f_o_F_2_ and f_o_F_1_ for solar minimums and maximums are given in a comparison with the IRI-2016 model (dashes). Solid curves are polynomial approximations. Points from 0°–40°E are repeated at 360°–400°E longitudes. Arrows indicate the longitude of the geomagnetic pole meridian.

The reduced f_o_F_2_ and f_o_F_1_ are given in Fig. 1 in comparison to the IRI-2016 (https://ccmc.gsfc.nasa.gov/modelweb/models/iri2016_vitmo.php) model variations. Pronounced f_o_F_2_ and f_o_F_1_ longitudinal variations are observed under both levels of solar activity. The IRI-2016 model describes the observed f_o_F_2_ with sufficient accuracy, while the f_o_F_1_ model values are overestimated under solar minimum.

The interpolated positions of maxima at 50°–60°E and minima at 240°–250°E are very close for f_o_F_2_ and f_o_F_1_ (Fig. 1), but they do not coincide with the longitude of the magnetic pole or the λ_pol_ and (λ_pol_ + 180^0^) longitudes. The extrema are shifted to the west with respect to the magnetic pole meridian. Ionospheric F_1_ and F_2_ layers have different formation mechanisms, but they both depend on the same neutral composition, and the coincidence of extreme positions confirms the controlling role of neutral composition in longitudinal variations.

**Figure 2:**
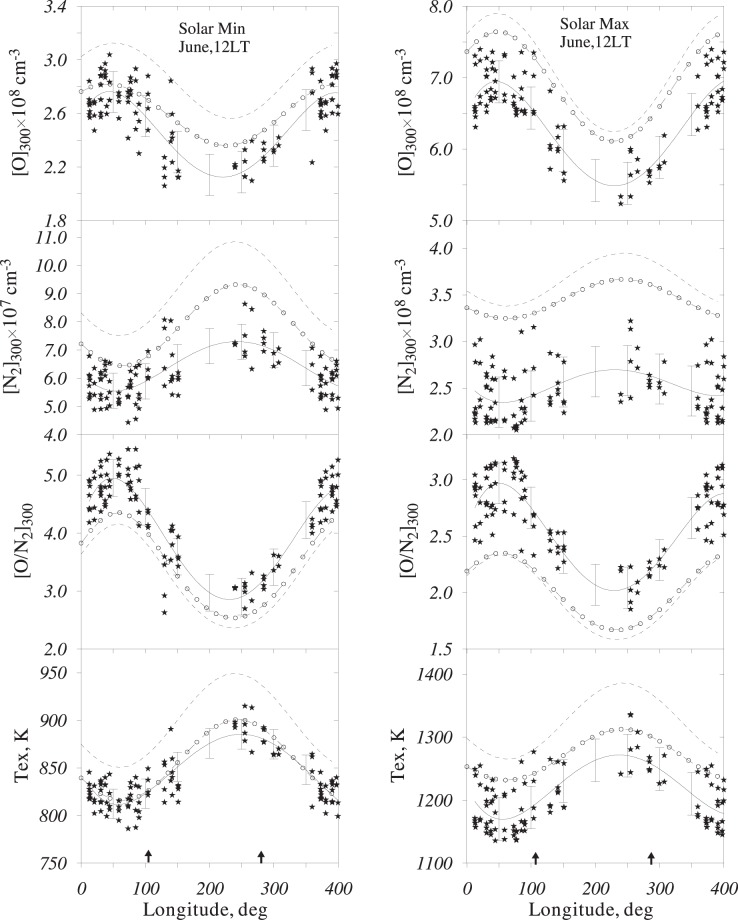
Longitudinal variations in the inferred thermospheric parameters at 300 km and 50°N for years with a solar minimum and maximum. Solid lines – polynomial approximations with error bars (SD values are given); dashes – MSIS-86 model; and circles – MSISE-00 model. Points from 0°–40°E are repeated at 360°–400°E longitudes. Arrows indicate the longitude of the geomagnetic pole meridian.

The application of method^10^ to ionospheric observations at 26 stations has given us neutral temperature and composition at F-region heights (>140 km). The retrieved neutral composition and temperature were reduced to the same latitude of 50°N and fixed levels of solar activity using the MSIS-86 model^17^ internal structure. The input June monthly F_10.7_ and Ap indices averaged over the years of solar minimum are F_10.7_ = 71 and Ap = 10 nT and F_10.7_ = 167 and Ap = 11 nT for years with a solar maximum. When reduced this way, [O], [N_2_], O/N_2_ ratio, and Tex are given in Fig. 2 for the solar minimum and maximum in a comparison to thermospheric models MSIS-86 and MSISE-00.

Both the retrieved and modelled values manifest pronounced longitudinal variations (Fig. 2). The extrema are located at 50°–60°E and 240°–250°E with similar f_o_F_1_ and f_o_F_2_ variations (Fig. 1). This coincidence is not surprising, as daytime f_o_F_2_ and f_o_F_1_ reflect corresponding variations in the thermospheric parameters. The extrema in Fig. 2 are also shifted to the west with respect to the magnetic pole meridian. In general, MSISE-00 (which has nothing in common with the retrieval method) is closer to the retrieved variations in thermospheric parameters compared to MSIS-86. Although the model and retrieved longitudinal variations appear very similar, the absolute differences are also observed. Modelled Tex values are systematically larger than the inferred ones, especially with MSIS-86. This results in larger [N_2_] concentrations, especially in the American longitudinal sector. It is interesting to note that despite noticeable differences in Tex, [N_2_], and [O] between the two versions of the MSIS model, the longitudinal variations in the O/N_2_ ratio are very similar (Fig. 2).

The similarity between the retrieved and modelled longitudinal variations in thermospheric parameters looks interesting, as the compared variations were obtained with very different methods using very different source data. This similarity is also confirmed by the relative (maximum/minimum ratio) variations given in Table 2. Perfect coincidence is observed for the O/N_2_ ratio under both levels of solar activity and for other parameters under solar maximum conditions. The largest difference occurs for atomic oxygen under a solar minimum when MSISE-00 underestimates the magnitude of [O] longitudinal variations (also Fig. 2). This is mainly due to lower [O] values in the American longitudinal sector.

**Table 2:** Magnitudes of longitudinal variations for the retrieved and modelled thermospheric parameters at 300 km, 50°N, and 12 LT in June during solar minimum and maximum conditions.

Parameter	Solar minimum			Solar maximum		
	Retrieved	MSIS-86	MSISE-00	Retrieved	MSIS-86	MSISE-00
[O]	1.30	1.22	1.19	1.27	1.27	1.25
[N_2_]	1.31	1.44	1.45	1.15	1.17	1.13
O/N_2_	1.73	1.76	1.71	1.47	1.47	1.41
T_ex_	1.09	1.12	1.10	1.09	1.09	1.06

## Discussion

From the very beginning, the mechanism of longitudinal/UT variations in neutral composition has been associated with high-latitude heating and displacement between the geomagnetic and geographic poles^14,18,19^. Due to Joule and particle precipitation heating in the auroral zone, the upper atmosphere expands, and this upwelling results in a decrease in the O/N_2_ ratio at a fixed height. Equatorward solar driven and/or disturbed thermospheric circulation transfers this disturbed neutral composition to lower latitudes. This mechanism has been discussed in the literature^20–22^. The near-to-pole longitudinal (American) sector should manifest larger [N_2_] and lower [O] and O/N_2_ compared to the European sector at the same geographic latitudes, as shown in Fig. 2. The reduction in the retrieved thermospheric parameters at the same geomagnetic latitude Ф = 50° (not shown in the paper) only slightly changes the pattern of longitudinal variations, shifting the extrema farther to the west.

One may conclude that June auroral heating is systematically larger in the American sector. A plausible explanation for this extra heating is the larger Joule heating due to the larger conductivity in the auroral zone. The auroral oval (http://www.sws.bom.gov.au/Aurora/3/1) receives more sunlight in June in the American sector than in the European sector. The noontime solar zenith angle χ is 42° at the longitude of the magnetic pole (73°W), but noontime χ is 61° at the antipode longitude of 107°E. Considering the electron concentration in the E-region^23^ N_m_E∼(cosχ)^0.6^, the expected difference in the electron concentration is ∼30%, which provides a larger conductivity.

A westward shift in the extrema of the longitudinal variations with respect to the longitude of the magnetic pole meridian taking place both in the ionospheric (Fig. 1) and retrieved parameters, as well as in the modelled thermospheric parameters (Fig. 2), reveals the reality of this shift, which may be related to dominating westward circulation at mid-latitudes during the June solstice^24^. A westward tilt was also observed in the mean thermospheric mass density^11^.

Longitudinal variations in the daytime column O/N_2_ ratio from TIMED/GUVI observations on solstices were analysed by the authors^12^. The column O/N_2_ ratio in those observations^25^ was calculated above the level where the column N_2_ abundance of 10^17^ cm^−2^ was located at a 147–150 km height. It is interesting to compare the observed column O/N_2_ ratio to our retrieved and MSISE-00 modelled longitudinal/solar activity variations. For this comparison, we selected stations with close geographic latitudes, Rome (41.9°N, 12.5°E; Φ = 42°) and Boulder (40.0°N, 254.7°E; Φ = 48.5°), and a pair of stations with close geomagnetic latitudes, Juliusruh (54.6°N, 13.4°E; Φ = 54°) and Millstone Hill (42.5°N, 288.5°E; Φ = 53.3°), which are located in the European and American longitudinal sectors, respectively. The last deep solar minimum in 2009 (F_10.7_ = 68.6; Ap = 4.1) and solar maximum in 2000 (F_10.7_ = 179.8; Ap = 15.2) were taken for our analysis, where F_10.7_ and Ap are June monthly indices. Observed June noontime monthly median N_m_F_2_ values are given in a comparison to IRI-2016 values to show that the selected stations manifest N_m_F_2_ similar to the modelled results. (Table 3)

**Table 3:** Observations and IRI model results of June noontime monthly median N_m_F_2_ for solar minimum (2009) and maximum (2000) years.

Years	Stations	N_m_F_2_ × 10^5^, cm^−3^	N_m_F_2_ × 10^5^, cm^−3^ (IRI)
2009	Rome	3.22	3.51
	Juliusruh	2.87	2.74
	**Boulder**	**2.86**	**2.87**
	**Millst. Hill**	**2.62**	**2.87**
2000	Rome	10.71	9.56
	Juliusruh	6.76	6.43
	**Boulder**	**5.78**	**6.54**
	**Millst. Hill**	**6.60**	**6.07**

The observed monthly median f_o_F_2_ and f_o_F_1_ at the four stations were used to retrieve thermospheric parameters and calculate column O/N_2_ ratios above the level with a column N_2_ abundance of 10^17^ cm^−2^, as was done in the observations^12^. Table 4 gives a comparison with the MSISE-00 modelled column O/N_2_ ratios.

**Table 4:** Inferred and MSISE-00 model (in parentheses) height-integrated June noontime O/N_2_ ratios in the European and American sectors under solar maximum (2000) and minimum (2009) monthly median conditions.

Station	2009	2000
Rome	0.632 (0.551)	0.802 (0.701)
Juliusruh	0.514 (0.476)	0.682 (0.588)
Boulder	0.554 (0.460)	0.696 (0.606)
Millst. Hill	0.507 (0.465)	0.694 (0.598)

Table 4 shows that both the inferred and MSISE-00 modelled height-integrated O/N_2_ ratios increase with solar activity. Our results and the MSISE-00 values are comparable with the TIMED/GUVI observations^12^ at 12 LT in June under solar minimum conditions at ∼45°N with a column O/N_2_ ratio ∼ 0.5. However, the TIMED/GUVI observations manifest the inverse dependence on solar activity, and the observed height-integrated O/N_2_ ratio is <0.4 at ∼45°N under high solar activity^12^ (their Fig. 4), while our inferred and MSISE-00 column O/N_2_ ratios are 0.6–0.8 in 2000 (Table 4). On the other hand, qualitatively TIMED/GUVI observations demonstrate correct longitudinal variations with a larger column O/N_2_ ratio in the European sector compared to the American sector in accordance with our results and the MSISE-00 model results.

The increase in the retrieved and MSISE-00 modelled height-integrated O/N_2_ ratio with solar activity indicates an increase in atomic oxygen abundance under solar maximum conditions. This is determined from the following. Above the turbopause, which is located at 110–120 km (while the level with column N_2_ content of 10^17^ cm^−2^ is at ∼150 km), the neutral species are distributed in accordance with the barometric law; therefore, the column content of any species above the height h is N_h_H, where N_h_ is the concentration and H is the scale height kT/mg of a given species. Therefore, the O/N_2_ column ratio is independent of neutral gas temperature but depends only on the [O]/[N_2_] ratio at a fixed height h. Atomic oxygen is completely produced and lost in the upper atmosphere^26^, forming a layer with a maximum at ∼97 km and zero concentrations below 80 km^27^. Therefore, height-integrated [O] above 70 km gives the total column content of atomic oxygen. Table 5 gives the MSISE-00 modelled total column contents of [O] and [N_2_] above 70 km under solar maximum (2000) and solar minimum (2009) conditions, in addition to Tex and neutral temperature at a 70 km height, at four locations.

**Table 5:** MSISE-00 modelled total column contents of [O] and [N_2_] above 70 km under solar maximum (2000, first line) and solar minimum (2009, second line) conditions, in addition to Tex and neutral temperature at a 70 km height, at four locations.

Parameter	Rome	Boulder	Juliusruh	Millstone Hill
[O]_col_ × 10^17^, cm^−2^	8.12 7.69	7.13 6.54	6.39 6.14	6.90 6.45
[N_2_]_col_ × 10^20^, cm^−2^	9.81 9.79	9.65 9.63	10.96 10.93	9.84 9.82
Tex, K	1265 781	1308 817	1296 805	1312 816
T_70_, K	209 209	210 210	210 210	209 209

Table 5 shows a 4–9% increase in the [O] column content in 2000 compared to 2009, while [N_2_]_col_ is practically unchanged. The increase in the atomic oxygen abundance under high solar activity may be attributed to an increase in the intensity of the Schumann-Runge continuum, which is responsible for the dissociation of O_2_ in the upper atmosphere. The stability of the MSISE-00 modelled [N_2_]_col_ under varying solar activity is due to relatively stable neutral temperatures at mesospheric heights, which provide the main contribution to the [N_2_] column content (Table 5).

The TIMED/GUVI height-integrated O/N_2_ ratios are sometimes used to interpret global-scale seasonal and solar activity N_m_F_2_ variations^13^. Indeed, a simplified formation mechanism of the mid-latitude daytime F_2_-layer, ignoring vertical plasma drift, may be related to the O/N_2_ ratio taken at the F_2_-layer maximum height^28^

$${N}_{m}{F}_{2}=0.75\frac{{q}_{m}}{{\beta }_{m}}$$

where q_m_ is the O^+^ ion production rate and β_m_ is the linear loss coefficient taken at h_m_F_2_. With some reservations, q_m_/β_m_ may be considered to be proportional to (O/N_2_)_max,_ but this ratio taken at h_m_F_2_ is not the same as the height-integrated O/N_2_ ratio. Our method^10^ provides the necessary h_m_F_2_ to calculate (O/N_2_)_max_. Table 6 gives the Rome/Millstone Hill and Rome/Boulder ratios for the observed N_m_F_2_, (O/N_2_)_max_ and (O/N_2_)_col_ ratios for the two levels of solar activity.

**Table 6:** Rome/Millstone Hill and Rome/Boulder ratios for observed N_m_F_2_, retrieved O/N_2_ ratio at h_m_F_2_, and column O/N_2_ ratio calculated from the retrieved [O] and [N_2_] for June 2009 and 2000.

Parameter	2009		2000	
	Rome/Mill. Hill	Rome/Boulder	Rome/Mill. Hill	Rome/Boulder
Δ(N_m_F_2_)_obs_	1.23	1.13	1.62	1.85
Δ(O/N_2_)_max_	1.27	1.00	1.49	1.97
Δ(O/N_2_)_col_	1.25	1.14	1.16	1.15

Table 6 shows that (O/N_2_)_col_ longitudinal variations are small (∼15%) in comparison with the observed N_m_F_2_ variations under high solar activity. They are close only during the deep solar minimum in 2009, while the (O/N_2_)_max_ longitudinal variations are much closer to the observed N_m_F_2_ variations under both solar activity conditions. This is not a surprise, as the level with a N_2_ column density of 10^17^ cm^−2^ (used to calculate the column O/N_2_ ratio) is located at heights of 147–150 km, i.e., much further below the F_2_-layer maximum; however, these concentrations provide the main contribution to the column density, but they do not participate in the F_2_-layer formation.

Another problem with using the column (O/N_2_) ratio to interpret any spatial, seasonal, or solar activity N_m_F_2_ variations is the smoothing temperature effect. The atomic oxygen concentration is a crucial parameter for F_2_-region formation as N_m_F_2_∼[O]^4/3^ during daytime hours^29^. Its concentration in the American sector is 30% less than that in the Eurasian sector (Table 2), and this difference is mainly responsible for the observed N_m_F_2_ longitudinal variations. However, T_ex_ and, correspondingly, the atomic oxygen scale height are larger in the American sector (Fig. 2), which decreases the difference in the height-integrated O/N_2_ ratios between the two sectors. Keeping all of this in mind, one may conclude that the column O/N_2_ ratio cannot be used for any quantitative interpretation of N_m_F_2_ variations.

## Conclusions

The obtained results are summarized as follows.

1. The observed longitudinal f_o_F_1_ and f_o_F_2_ variations are similar to the retrieved and MSIS modelled thermospheric parameter variations, indicating their general consistency. The best coincidence with the empirical models is related to the inferred O/N_2_ ratio, while MSISE-00 underestimates the magnitude of [O] longitudinal variations under solar minimum conditions. In general, similar variations in thermospheric parameters obtained with different methods and different data sources are interesting.
2. The American sector manifests larger Tex values (independent of both the geographic and geomagnetic latitudes considered) under both solar maximum and minimum conditions. A plausible explanation for this extra heating is the larger conductivity in the auroral oval, which receives more sunlight in June in the American sector compared to the European sector.
3. A westward shift in the extreme position in terms of longitudinal variations with respect to the longitude of the magnetic pole meridian, taking place both for ionospheric and thermospheric parameters, may be related to dominating westward circulation at mid-latitudes during the June solstice^24^.
4. The inferred and MSISE-00 height-integrated O/N_2_ ratios are comparable to the TIMED/GUVI observations only under solar minimum conditions, with a column O/N_2_ ratio ∼ 0.5 at 12 LT in June at ∼45°N^12^ (their Fig. 2). However, the TIMED/GUVI observations manifest an inverse dependence on solar activity with a height-integrated O/N_2_ ratio < 0.4 under high solar activity, which is contrary to the retrieved and MSISE-00 modelled column O/N_2_ ratios (0.6–0.8).
5. The retrieved height-integrated (O/N_2_) ratio longitudinal variations are small (∼15%) in comparison with the observed N_m_F_2_ variations under high solar activity. A 30% difference in atomic oxygen concentration between the American and European sectors is mainly responsible for the observed N_m_F_2_ longitudinal variations and is strongly compensated in (O/N_2_)_col_ by a larger Tex in the American sector. The height-integrated O/N_2_ ratio cannot be incorporated into the F_2_-layer formation mechanism; therefore, such observations cannot be used for any quantitative interpretation of N_m_F_2_ variations.
